# Analysis of dynamic molecular networks for pancreatic ductal adenocarcinoma progression

**DOI:** 10.1186/s12935-018-0718-5

**Published:** 2018-12-22

**Authors:** Zongfu Pan, Lu Li, Qilu Fang, Yiwen Zhang, Xiaoping Hu, Yangyang Qian, Ping Huang

**Affiliations:** 1 Department of Pharmacy, Zhejiang Cancer Hospital, Hangzhou, 310022 China; 2 Department of Pharmacy, The First Affiliated Hospital, College of Medicine, Zhejiang University, Hangzhou, 310003 China; 3 Key Laboratory of Head & Neck Cancer Translational Research of Zhejiang Province, Zhejiang Cancer Hospital, Hangzhou, 310022 China

**Keywords:** Dynamic molecular networks, Pancreatic ductal adenocarcinoma, Progression, Bioinformatics

## Abstract

**Background:**

Pancreatic ductal adenocarcinoma (PDAC) is one of the deadliest solid tumors. The rapid progression of PDAC results in an advanced stage of patients when diagnosed. However, the dynamic molecular mechanism underlying PDAC progression remains far from clear.

**Methods:**

The microarray GSE62165 containing PDAC staging samples was obtained from Gene Expression Omnibus and the differentially expressed genes (DEGs) between normal tissue and PDAC of different stages were profiled using R software, respectively. The software program Short Time-series Expression Miner was applied to cluster, compare, and visualize gene expression differences between PDAC stages. Then, function annotation and pathway enrichment of DEGs were conducted by Database for Annotation Visualization and Integrated Discovery. Further, the Cytoscape plugin DyNetViewer was applied to construct the dynamic protein–protein interaction networks and to analyze different topological variation of nodes and clusters over time. The phosphosite markers of stage-specific protein kinases were predicted by PhosphoSitePlus database. Moreover, survival analysis of candidate genes and pathways was performed by Kaplan–Meier plotter. Finally, candidate genes were validated by immunohistochemistry in PDAC tissues.

**Results:**

Compared with normal tissues, the total DEGs number for each PDAC stage were 994 (stage I), 967 (stage IIa), 965 (stage IIb), 1027 (stage III), 925 (stage IV), respectively. The stage-course gene expression analysis showed that 30 distinct expressional models were clustered. Kyoto Encyclopedia of Genes and Genomes analysis indicated that the up-regulated DEGs were commonly enriched in five fundamental pathways throughout five stages, including pathways in cancer, small cell lung cancer, ECM-receptor interaction, amoebiasis, focal adhesion. Except for amoebiasis, these pathways were associated with poor PDAC overall survival. Meanwhile, LAMA3, LAMB3, LAMC2, COL4A1 and FN1 were commonly shared by these five pathways and were unfavorable factors for prognosis. Furthermore, by constructing the stage-course dynamic protein interaction network, 45 functional molecular modules and 19 nodes were identified as featured regulators for all PDAC stages, among which the collagen family and integrins were considered as two main regulators for facilitating aggressive progression. Additionally, the clinical relevance analysis suggested that the stage IV featured nodes MLF1IP and ITGB4 were significantly correlated with shorter overall survival. Moreover, 15 stage-specific protein kinases were identified from the dynamic network and CHEK1 was particularly activated at stage IV. Experimental validation showed that MLF1IP, LAMA3 and LAMB3 were progressively increased from tumor initiation to progression.

**Conclusions:**

Our study provided a view for a better understanding of the dynamic landscape of molecular interaction networks during PDAC progression and offered potential targets for therapeutic intervention.

**Electronic supplementary material:**

The online version of this article (10.1186/s12935-018-0718-5) contains supplementary material, which is available to authorized users.

## Background

Pancreatic ductal adenocarcinoma (PDAC) is one of the most malignant solid tumors arising within the ductal of the pancreas. The lack of early diagnosis and its rapid progression resulted in an advanced stage of PDAC patients when diagnosed. In the past several decades, many efforts have been taken to unveil the molecular pathogenesis of PDAC, and to improve the patient prognosis through various therapeutic strategies. However, limited advances have been made to prolong the survival and to reduce the mortality. The cancer staging is one of the most important factors in determining treatment strategies and predicting a patient’s outcome [1]. At present, surgical resection is the standard care for only 20% of patients with localized disease, while most patients with surgical management will develop cancer recurrence and die within 2 years. Moreover, the median survival of advanced inoperable PDAC patient with systemic chemotherapy is only about 8 months [2]. Therefore, exploring the molecular mechanism underlying the progression of PDAC may contribute to the development of precise therapeutics for PDAC patients.

PDAC is a complex disease driven by time and context dependent alterations of multiple genes. Considerable advances have been made in demonstrating the genetic alterations involved in the progression PDAC. Frequent mutations in Kirsten Ras (K-RAS), TP53, CDKN2A and SMAD4 has been identified as essential drivers for PDAC development [3]. According to the integrated genomic analysis, Bailey et al. [4] revealed different mechanism of molecular evolution underlying four redefined pancreatic cancer subtypes, which provides a new insight into potential therapeutic relevance and patient selection. Depending on the spatiotemporal proteomic analysis of pancreas cancer progression, Mirus et al. [5] pointed out that dynamic expression pattern of serine/threonine stress kinase 4 were associated with early tumorigenic events. Additionally, genome-wide transcriptome analysis by Jones et al. [6] showed that more than 21,000 genetic altered in PDAC, which mainly affected 12 core signaling pathways including apoptosis, DNA damage repair, cell adhesion and invasion. Besides, Janky et al. [7] screened the master regulators of transcription involved in PDAC progression and highlighted the HNF1A/B as a putative tumor suppressor in pancreatic cancer. However, despite these important advances, the precise dynamic landscape of molecular interaction networks during PDAC progression is incompletely understood.

The molecular interactions in tumor are varying with cancer staging. Construction and analysis of dynamic molecular networks provide a view to understand dynamic cellular mechanisms of different biological process during cancer progression and offer opportunities for therapeutic intervention. In the present study, we analyzed the microarray from Gene Expression Omnibus and identified the differentially expressed genes between normal tissue and PDAC of different stages, respectively. Further, we constructed the dynamic protein–protein interaction networks and analyzed different topological variation of nodes and clusters over time. Several functional molecular modules and genes were identified as key regulators for PDAC development. Through visualizing the dynamic networks from early to advanced stage, our study aimed at improving our understanding of the underlying mechanism of PDAC progression and provided novel insights for precise treatment.

## Methods

### Tissue samples

Human PDAC tissues with four different stages and non-tumorous tissues were obtained from five patients who underwent surgical resection at Zhejiang Cancer Hospital. All specimens had a pathological diagnosis at the time of assessment. Studies were approved by the Ethics Committee of Zhejiang Cancer Hospital.

### Microarray data

Microarray dataset GSE62165 containing staging pancreatic ductal adenocarcinoma (PDAC) and non-tumoral pancreatic tissue was obtained from Gene Expression Omnibus (GEO, http://www.ncbi.nlm.nih.gov/geo/) database in the National Center for Biotechnology Information (NCBI), which was deposited by Janky and colleague [7]. The GSE62165 dataset included 118 surgically resected PDAC varying from stage I to stage IV and 13 control samples. The detail PDAC patient cohort consisted of 8 stage I samples, 30 stage IIa samples, 62 stage IIb samples, 5 stage III samples and 13 stage IV samples. Additionally, the gene expression profile was detected basing on HG-U219 (Affymetrix Human Genome U219 Array) platform.

### Data processing and screening of differentially expressed genes (DEGs)

The CEL file data of GSE62165 was read using the affy package in the R language software (version 3.2.3, https://www.r-project.org/). Background correction, normalization, expression calculation of the original array data and log2 transformation were processed by robust multichip average (RMA) algorithm. Empirical Bayes method was used to identify significant DEGs between different stages of PDAC and normal pancreatic samples basing on the limma package in R. *P* values were adjusted for multiple testing depending on the Benjamini–Hochberg False Discovery Rate (FDR) method. The strict thresholds for identifying DEGs were set as FDR < 0.01 and |log2 fold change (FC)| ≥ 2.

### Analysis of DEGs expression manner during PDAC progression

The software program Short Time-series Expression Miner (STEM) is designed for clustering, comparing, and visualizing gene expression data from short time series microarray experiments [8]. To search the differences of gene expression between PDAC stages, the STEM tool was applied to profile candidate genes simultaneously from normal status to stage IV. Briefly, the union of DEGs from five stages was input and normalized by STEM. The maximum number of model profiles was set as 100, and FDR was selected as correction method. The rest parameters were remained unchanged. Colored clusters indicated statistically significant number of genes assigned.

### Function annotation and pathway enrichment of DEGs

Gene ontology (GO) and Kyoto Encyclopedia of Genes and Genomes (KEGG) were applied for the functional annotation and pathway enrichment analysis of DEGs through using the Database for Annotation Visualization and Integrated Discovery (DAVID; https://david.ncifcrf.gov/) [9–11]. Adjusted *P* values were calculated by Benjamini–Hochberg FDR method and the thresholds were set as FDR < 0.05 to indicate a statistically significant difference.

### Dynamic protein–protein interaction (PPI) network construction and variation analysis of node centrality and cluster

The online database Search Tool for the Retrieval of Interacting Genes (STRING, http://string-db.org/) is commonly used to identify the interactions between known proteins and predict proteins and to construct a PPI network [12]. The DEGs were input into STRING to construct PPI network and further visualized by Cytoscape (version 3.6.1) software. The Cytoscape plugin DyNetViewer was applied to construct the stage-course dynamic protein interaction network [13]. The time course protein interaction networks (TC-PIN) algorithm was chosen to constructing sub-networks and the threshold was set as 2. For dynamic node centrality analysis, three typical centrality measures including degree centrality (DC), betweenness centrality (BC), local average connectivity-based method (LAC) were used. The top 20 DC, BC, and LAC of genes that uniquely elevated at each stage were firstly identified, respectively. Then, the common genes among the top 20 DC, BC, and LAC lists at each stage were considered as key nodes for dynamic network development. For dynamic module analysis, clustering algorithm molecular complex detection (MCODE) was conducted for analyzing clusters of dynamic networks. The degree cutoff was set as 5 and K-core was set as 2.

### Identification of PDAC stage-specific activated kinases and phosphosite markers

To identify the stage-specific activated kinase during PDAC progression, total protein kinase list was acquired from the human kinome database (kinase.com) [14] and was intersected with DEGs. Then, the dynamic DC, BC and LAC of intersection kinases were retrieved from the results of dynamic node centrality analysis described above. Furthermore, the substrates of kinases were searched from PhosphoSitePlus (https://www.phosphosite.org) online tool. The sequence characteristics of kinase substrates were depicted by sequence logo to indicate the phosphosite markers.

### Kaplan–Meier survival analysis of key genes in PDAC

The online database Kaplan–Meier plotter (www.kmplot.com) is capable to retrieve gene expression data and clinical information from GEO, European Genome-phenome Archive (EGA) and The Cancer Genome Atlas (TCGA) [15]. To evaluate the prognostic value of candidate genes, the patient samples were split into two cohorts according to the best cutoff of gene expression computed by Kaplan–Meier plotter. To analyze the relationship between particular pathways and overall survival, the mean expression of pathway associated signature genes and best cutoff were calculated by Kaplan–Meier plotter. Besides, the log rank *P* value and hazard ratio (HR) with 95% confidence intervals were also computed.

GEPIA is a newly developed interactive web server for estimating the RNA sequencing expression data from the TCGA and Genotype-Tissue Expression (GTEx) dataset projects [16]. Candidate genes were queried by GEPIA to explore their expression levels at different stages using TCGA-pancreatic adenocarcinoma data.

### Immunohistochemistry

Paraffin-embedded sections were deparaffinized and rehydrated firstly. Then, 1 mM EDTA (pH 8.0) was applied to retrieve the antigen. Endogenous peroxide activity was block by 0.3% hydrogen peroxide. Before incubating with primary antibody, 5% goat serum in TBS was used to avoid non-specific binding. The primary antibodies contained rabbit anti-MFL1IP (1:100, Proteintech, USA), rabbit anti-LAMA3 (1:200, Abbkine, China) and rabbit anti-LAMB3 (1:200, Abbkine, China). After incubating with biotinylated secondary antibodies, sections were developed using DAB (Beyotime, China) and counterstained with hematoxylin.

## Results

### Identification of DEGs at different pancreatic ductal adenocarcinoma (PDAC) stages and analysis of DEGs expression patterns

**Fig. 1:**
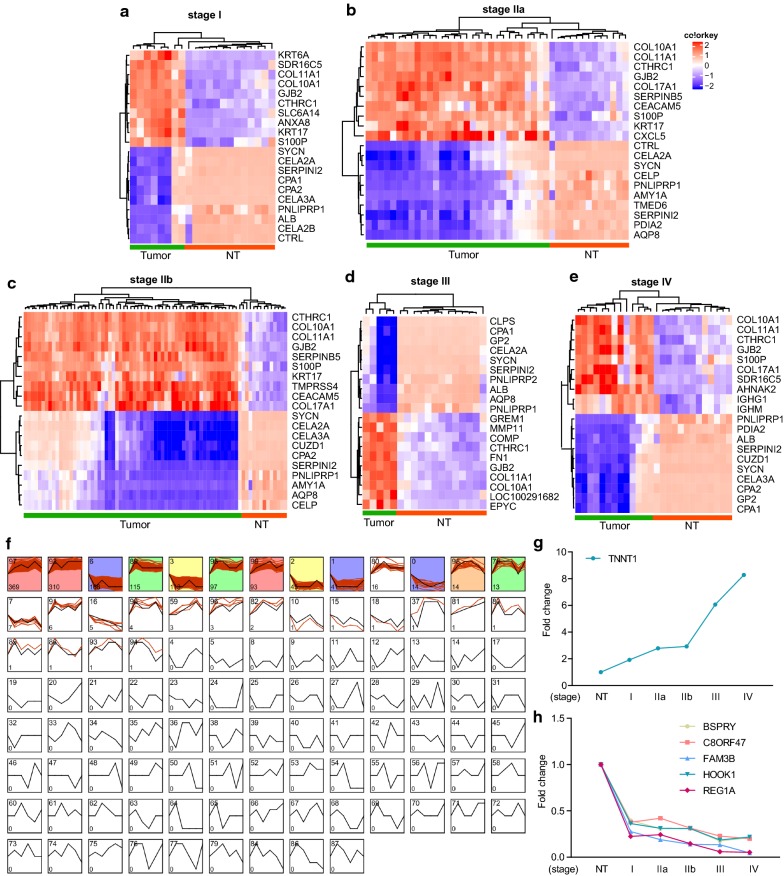
Identification and hierarchical clustering analysis of DEGs at different PDAC stages and the profiling of DEGs expression patterns. **a**–**e** Top 10 up and down-regulated DEGs of different stages were hierarchically clustered and displayed by heatmaps, respectively. **f** Gene expression patterns from normal tissue to PDAC stages were clustered, compared, and visualized by the STEM software. Colored clusters indicated statistically significant number of genes enriched. The number in bottom left indicated number of genes assigned. The number in the top left represented model identifier. **g**–**h** Classic gene expression trends were plotted from normal tissue (NT) to advanced PDAC stage

The microarray dataset GSE62165 was acquired from GEO. Differentially expressed genes (DEGs) (FDR < 0.01, |log2 FC| ≥ 2) of different PDAC stages were screened out basing on the R analysis, respectively. Compared with normal tissues, the total DEGs number for each stage were 994 (stage I), 967 (stage IIa), 965 (stage IIb), 1027 (stage III), 925 (stage IV). Then, the heatmaps of the top 10 up and down-regulated DEGs at each stage were hierarchically clustered and displayed, respectively (Fig. 1a–e). As the results showed in Fig. 1a–e, these top 20 DEGs could clearly distinguish each PDAC stage from normal pancreatic tissues.

To explore the differences of gene expression between PDAC stages, the STEM tool was applied to profile stage-course gene expression patterns. A total of 30 expression manners were enriched among 100 assumed expression models (Fig. 1f). Intriguingly, we found some clusters exhibited stage-specific expression trends throughout PDAC progression. Genes in the Model 89 (115 genes enriched) were specifically up-regulated at stage I. The Model 6 contained 166 genes and particularly elevated at stage IIa. The Model 3 (113 genes enriched) and Model 2 (45 genes enriched) were stage IIb- and stage III-specific gene clusters, respectively. Additionally, the Model 1 associated genes specifically increased at stage IV. These dynamic expression patterns suggested that distinct molecular signals were required for particular PDAC stages. Notably, we also found six genes that dramatically changed from normal status to stage IV (Fig. 1g–h). TNNT1, the troponin T type 1 encoding gene, was up-regulated increasingly from cancer initiation to advanced stage. Other five genes, including BSPRY, C8ORF47, FAM3B, HOOK1 and REG1A, were clustered in Model 16 and significantly decreased during cancer progression. These six genes may act as key regulators for driving PDAC progression via their special expression manners.

### Gene function annotation of DEGs at different stage

**Fig. 2:**
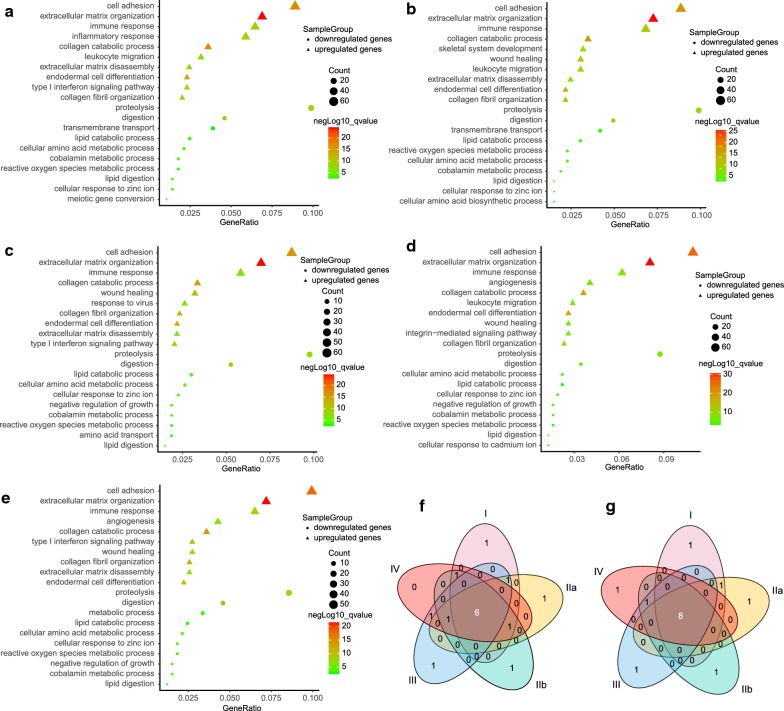
Top ten enriched GO (Biological Process) terms of up-regulated and down-regulated DEGs at different stages. **a**–**e** Gene function annotation of DEGs at stage I, IIa, IIb, III, and IV. **f** Venn diagram displayed common biological processes of up-regulated DEGs shared by five stages. **g** Venn diagram displayed common biological processes of down-regulated DEGs shared by five stages

To further investigate the biological function of DEGs between different PDAC stages and normal tissues, gene ontology (GO) analysis was performed using the DAVID online analysis tool. Basing on the results of GO biological process (BP) enrichment (Fig. 2a–f), we found up-regulated DEGs of five stages were commonly enriched in six biological processes, including cell adhesion, extracellular matrix organization, immune response, collagen fibril organization, collagen catabolic process, and endodermal cell differentiation. Moreover, extracellular matrix disassembly was shared by stage I, IIa, IIb, and IV. Notably, wound healing was enriched at stage IIa, IIb, III, and IV. Leukocyte migration was found in stage I, IIa, and III. Besides, type I interferon signaling pathway was commonly enriched by stage I, IIb, IV. Angiogenesis was particularly found in advanced PDAC including stage III and IV. Interestingly, inflammatory response was only found at early stage (stage I).

Functional annotation of down-regulated DEGs indicated that 8 biological processes were commonly enriched at each PDAC stage (Fig. 2a–e, g), including lipid digestion, cellular response to zinc ion, lipid catabolic process, reactive oxygen species metabolic process, cobalamin metabolic process, proteolysis, cellular amino acid metabolic process, digestion. Transmembrane transport was shared by stage I and IIa, while negative regulation of growth was conspicuously found at stage IIb, III, and IV. Additionally, meiotic gene conversion, cellular amino acid biosynthetic process, amino acid transport, cellular response to cadmium ion, and metabolic process were specifically enriched at each stage, respectively.

### Pathway enrichment of DEGs at different stages

**Fig. 3:**
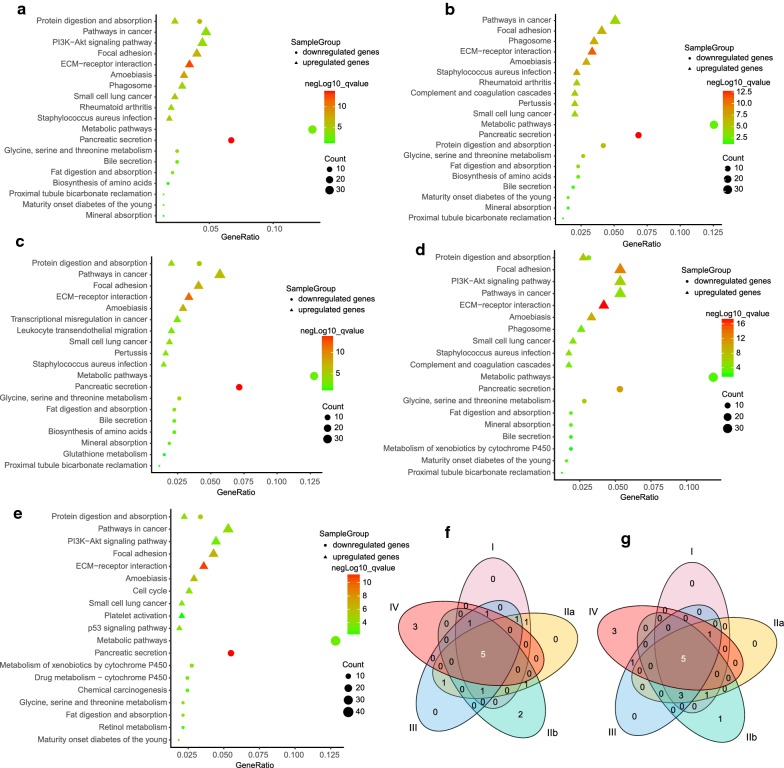
Top ten enriched KEGG pathways of up-regulated and down-regulated DEGs at different stages. **a**–**e** Pathway enrichment of DEGs at stage I, IIa, IIb, III, and IV, respectively. **f** Venn diagram displayed common pathways of up-regulated DEGs shared by five stages. **g** Venn diagram displayed common pathways of down-regulated DEGs shared by five stages

Subsequently, KEGG pathway analysis demonstrated that the up-regulated DEGs of different PDAC stages were commonly enriched in five key pathways including pathways in cancer, small cell lung cancer, ECM-receptor interaction, amoebiasis, focal adhesion (Fig. 3a–f). *Staphylococcus aureus* infection was enriched at every stage except stage IV. Phagosome was shared by stage I, IIa, III, while rheumatoid arthritis was only found at stage I and IIa. Protein digestion and absorption occurred at all stages except stage IIa. PI3K-Akt signaling pathway was enriched at stage I, III, and IV. Three pathways including p53 signaling pathway, platelet activation, and cell cycle were specifically enriched at stage IV.

The down-regulated DEGs of different PDAC stages were commonly enriched in five key pathways including metabolic pathways, glycine, serine and threonine metabolism, pancreatic secretion, protein digestion and absorption, fat digestion and absorption (Fig. 3a–e, g). Three pathways including mineral absorption, bile secretion, proximal tubule bicarbonate reclamation were found in all PDAC stages except stage IV. Biosynthesis of amino acids occurred at early stage (stage I, IIa, IIb). Maturity onset diabetes of the young was shared by stage I, IIb, III, IV. Metabolism of xenobiotics by cytochrome P450 was found in advanced PDAC at stage III and IV. For stage IV, three pathways were particularly enriched, including chemical carcinogenesis, drug metabolism—cytochrome P450, and retinol metabolism.

### Kaplan–Meier survival analysis of key pathways and signature genes

**Fig. 4:**
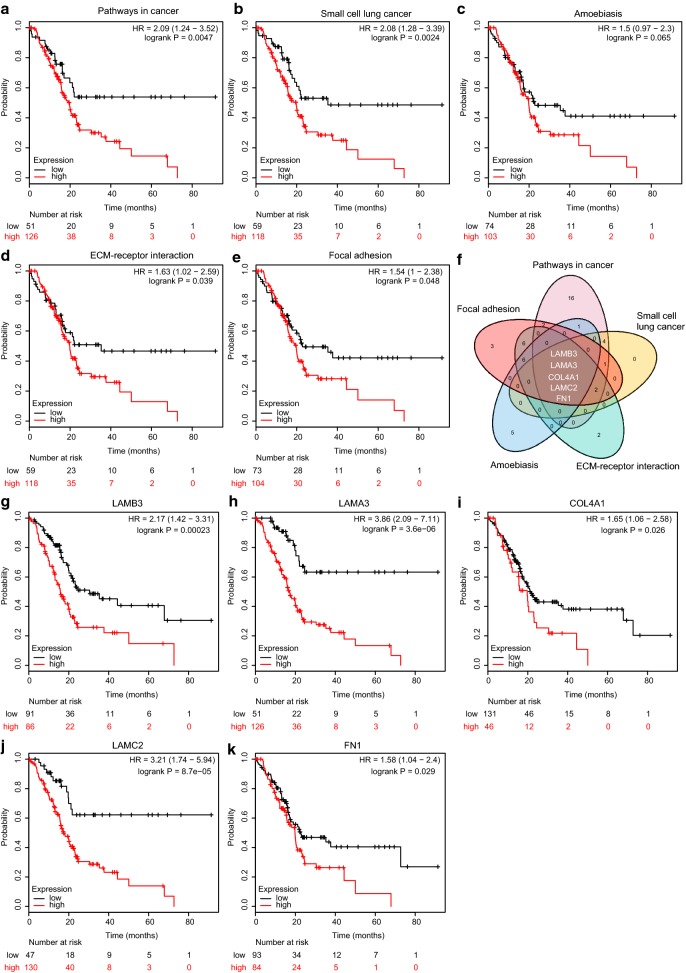
The clinical relevance of key pathways and signature genes. **a**–**e** Overall survival analysis of five fundamental pathways by Kaplan–Meier plotter. Log-rank test was used to evaluate significance. **f** Intersection of five fundamental pathways associated genes. **g**–**k** Overall survival analysis of five candidate genes by Kaplan–Meier plotter. Log-rank test was used to evaluate significance

To analyze the prognostic relevance of five key pathways shared by all PDAC stages, mean expression value of pathway associated genes and best cutoff were calculated by Kaplan–Meier plotter. As shown in Fig. 4a–e, pathways in cancer (*P *= 0.0047), small cell lung cancer (*P *= 0.0024), ECM-receptor interaction (*P *= 0.039) and focal adhesion (*P *= 0.048) were negatively associated with overall survival (OS). Moreover, the intersection of five pathways associated genes indicated that LAMB3, LAMA3, COL4A1, LAMC2 and FN1 were commonly enriched in these pathways (Fig. 4f). Kaplan–Meier survival analysis suggested that these five genes were unfavorable factors of prognosis (Fig. 4g–k).

### Protein–protein interaction (PPI) network construction

**Fig. 5:**
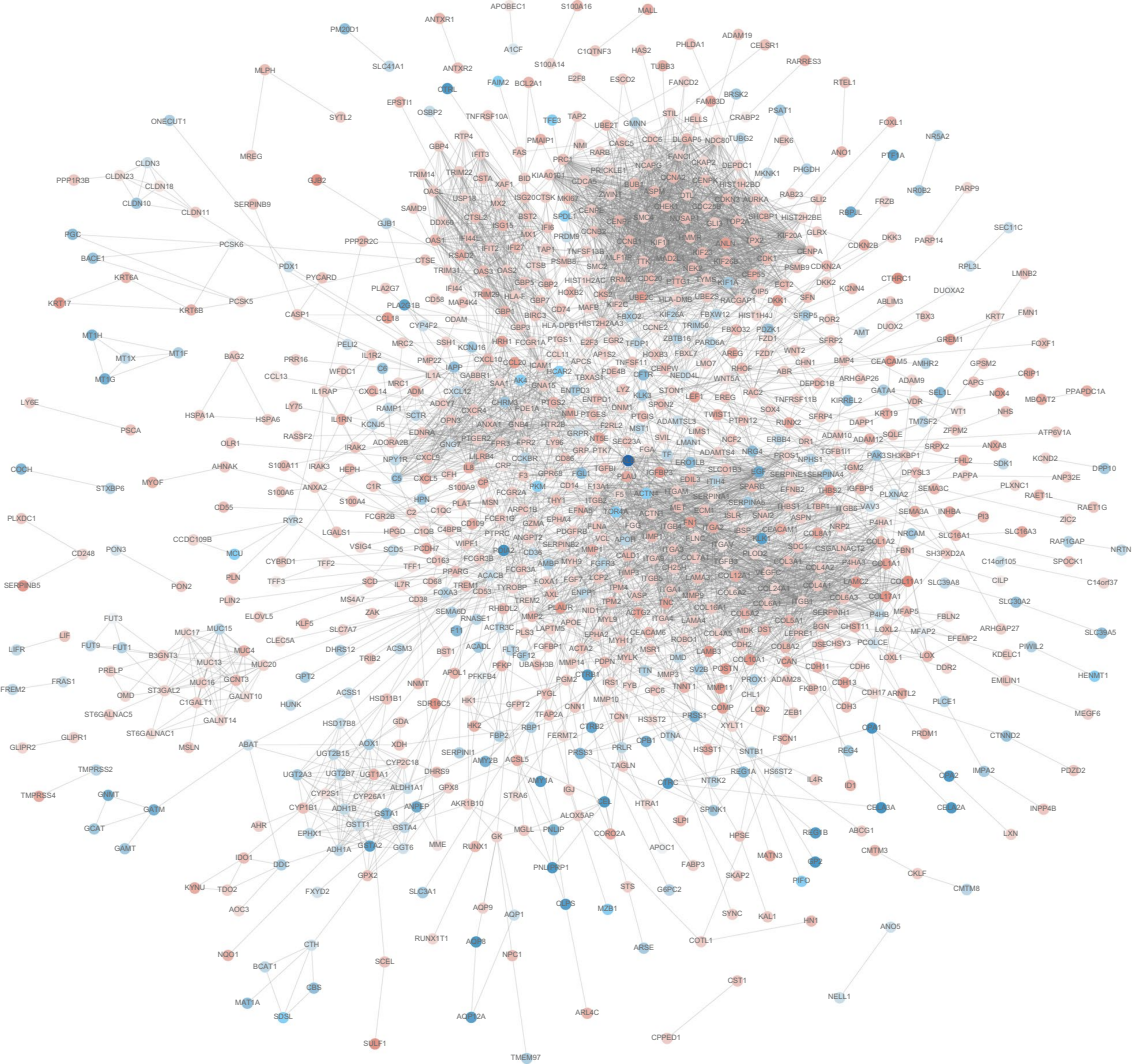
The protein–protein interaction network of DEGs at stage IV. The PPI network was constructed by STRING and visualized by Cytoscape software. Red nodes represented up-regulated DEGs. Blue nodes represented down-regulated DEGs

The PPI networks of the DEGs between PDAC and normal pancreatic tissues at different stages were constructed by the online database STRING. The typical PPI network of stage IV was shown in Fig. 5, and the network consisted of 868 nodes interacting via 4717 edges. Expression level of up-regulated DEGs and down-regulated DEGs in the PPI network was shown in red and blue.

### Cluster analysis of dynamic networks

**Fig. 6:**
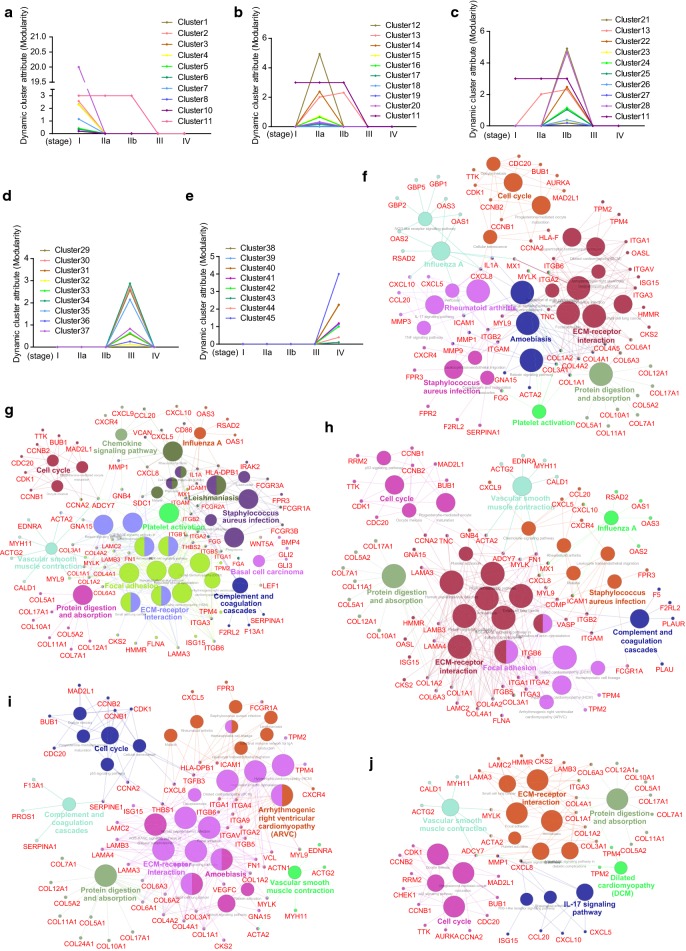
Clusters analysis of dynamic network during PDAC progression. **a**–**e** The charts of dynamic cluster attributes identified from dynamic networks at different stages. The dynamic cluster attributes for five stages were calculated by DyNetViewer (MCODE algorithm) over time. **f**–**j** Network of pathway analysis at each stage by ClueGO. Gene clusters of each stage were enriched for KEGG pathways and the interaction of pathways at five stages were further constructed by ClueGO, respectively

DyNetViewer is a novel Cytoscape app for constructing, analyzing, and visualizing dynamic molecular interaction networks. To analyze the dynamic cluster attribution for PDAC progression, the networks from five stages were input into DyNetViewer and calculated by MCODE algorithm over time. The clusters at each stage which exhibited highly interconnected regions were considered as key regulators for promoting networks development. A total of 45 featured molecular modules were identified for all PDAC stages (Additional file 1: Figures S1–S5). At stage I, 11 clusters that contributed to network were specifically screened out, among which the cluster 11 also contributed to the network of stage IIa and IIb (Fig. 6a). These 11 gene clusters were enriched in pathways of influenza A, cell cycle, rheumatoid arthritis, Staphylococcus aureus infection, amoebiasis, protein digestion and absorption, ECM-receptor interaction and platelet activation (Fig. 6f). Furthermore, we found that key genes including IL1A, CXCL8, ICAM1, ITGB2, ITGAM, COL1A1 and COL1A2 participated in as much as four pathways at stage I, respectively (Fig. 6f). Then, we found that ten clusters were particularly involved in the network of stage IIa, and the cluster 13 was also identified at stage IIb (Fig. 6b). Pathway enrichment showed that these clusters were mainly enriched in cell cycle, chemokine signaling pathway, influenza A, vascular smooth muscle contraction, focal adhesion, leishmaniasis, Staphylococcus aureus infection, protein digestion and absorption, ECM-receptor interaction, complement and coagulation cascades, basal cell carcinoma and platelet activation (Fig. 6g). CXCL8, ADCY7, ITGAM, ITGB2, ITGB1, IL1A, ICAM1, ITGA2, THBS2, SDC1, COL3A1, COL1A2 and COL1A1 were commonly shared by four or more pathways, respectively (Fig. 6g). In the network of stage IIb, ten clusters were found acting as key modules for the protein interaction network (Fig. 6c). These clusters were associated with cell cycle, vascular smooth muscle contraction, influenza A, Staphylococcus aureus infection, focal adhesion, complement and coagulation cascades, protein digestion and absorption and ECM-receptor interaction (Fig. 6h). ADCY7, ITGAM, ITGB2, MYL9 were shared by four or more pathways, respectively (Fig. 6h). For stage III, we found 9 unique clusters were essential for its network (Fig. 6d). These gene clusters mainly involved in cell cycle, vascular smooth muscle contraction, arrhythmogenic right ventricular cardiomyopathy, amoebiasis, complement and coagulation cascades, protein digestion and absorption and ECM-receptor interaction (Fig. 6i). Two key genes including CXCL8 and TGFB3 were shared by as much as four pathways, respectively (Fig. 6i). Moreover, a total of 8 clusters specifically participated in the network of stage IV (Fig. 6e). These clusters mainly enriched in cell cycle, vascular smooth muscle contraction, dilated cardiomyopathy, IL-17 signaling pathway, protein digestion and absorption and ECM-receptor interaction (Fig. 6j). No genes were found shared by more than two pathways at stage IV (Fig. 6j).

### Node centrality analysis of dynamic networks

**Fig. 7:**
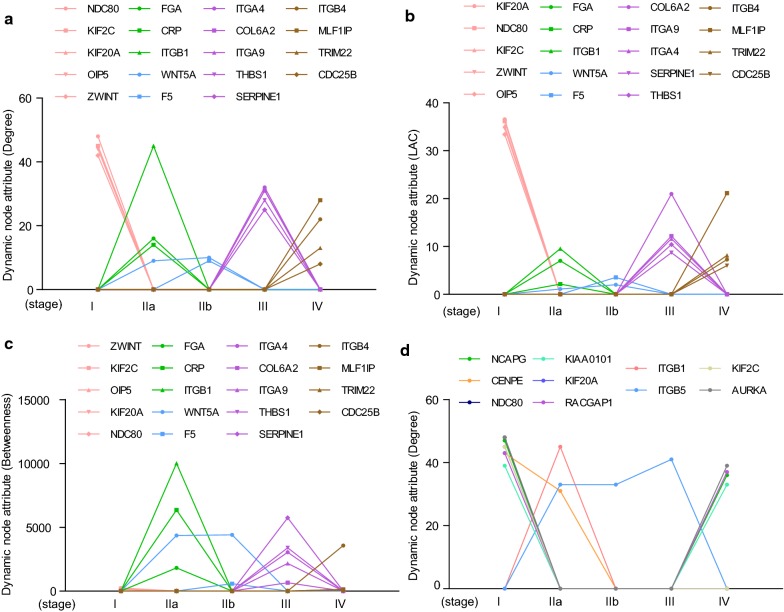
Node centrality of dynamic network during PDAC progression. **a**–**c** Featured nodes with values of DC, LAC and BC from dynamic networks at different stages. **b** The list of top ten nodes ranked by standard deviation of DC from dynamic network

To elucidated the key genes that participated in the dynamic networks, the node centrality at each PDAC stage were calculated. Three typical centrality measures including Betweenness Centrality (BC), Degree Centrality (DC), Local Average Connectivity-based method (LAC) were applied in our study. We screened out 19 key genes that potentially involved in the dynamic network development (Fig. 7a–c). Five genes including NDC80, KIF2C, KIF20A, OIP5, ZWINT were specifically found at stage I with highest DC, BC, and LAC. FGA, CRP and ITGB1 were featured nodes for stage II. WNT5A was essential for maintaining the network at stage IIa and IIb. F5 was uniquely found at stage IIb. Additionally, five genes including ITGA4, COL6A2, ITGA9, THBS1 and SERPINE1 were characteristic nodes at stage III. Four nodes including ITGB4, MLF1P, TRIM22 and CDC25B were potential key genes for promoting the advanced status of PDAC. Finally, we also sorted the top ten nodes ranked by standard deviation of DC, among which NCD80, KIF20A, ITGB1 and KIF2C also existed in the 19 key nodes mentioned above (Fig. 7d). The remaining genes including NCAPG, CENPE, KIAA0101, RACGAP1, ITGB5 and AURKA were also dynamically changed at particular stage (Fig. 7d).

### Kaplan–Meier survival analysis of key nodes

**Fig. 8:**
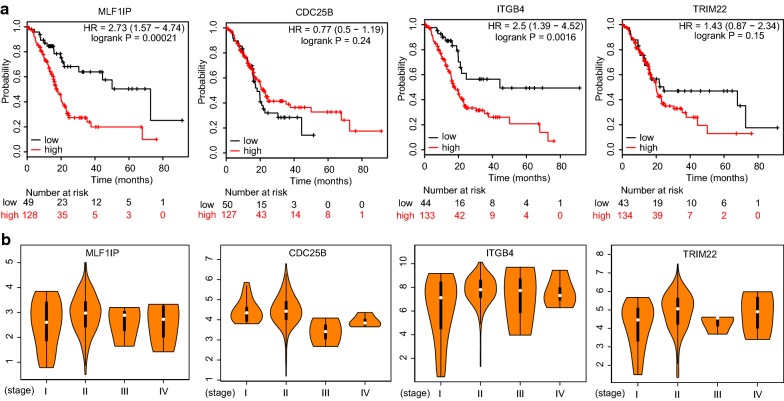
Kaplan–Meier curves for overall survival of key nodes. **a** Overall survival curves stratified by expression of MLF1IP, CDC25B, ITGB4 and TRIM22, respectively. Patients were divided into high-expression group and low-expression group, according to expression of four genes above. Log-rank test was used to evaluate significance. **b** Expression manners of MLF1IP, CDC25B, ITGB4 and TRIM22 at different stages were determined by GEPIA using TCGA-pancreatic adenocarcinoma data

According to the node centrality analysis above, we chose the featured nodes of stage IV to determine their clinical relevance. Kaplan–Meier survival analysis showed that high expression level of MLF1IP (also known as CENPU) and ITGB4 were significantly correlated with shorter overall survival, respectively (Fig. 8a). Furthermore, we also analyzed the expression manner of featured nodes (MLF1IP/CDC25B/ITGB4/TRIM22) during PDAC progression (Fig. 8b). The expression level of these four genes at stage IV showed no significant changes compared with other stages, which indicated that these nodes potentially promoted cancer progression by directly maintaining the molecular network without depending on their expression level (Fig. 8b).

### Identification of PDAC stage-specific activated protein kinases and the phosphosite markers

**Fig. 9:**
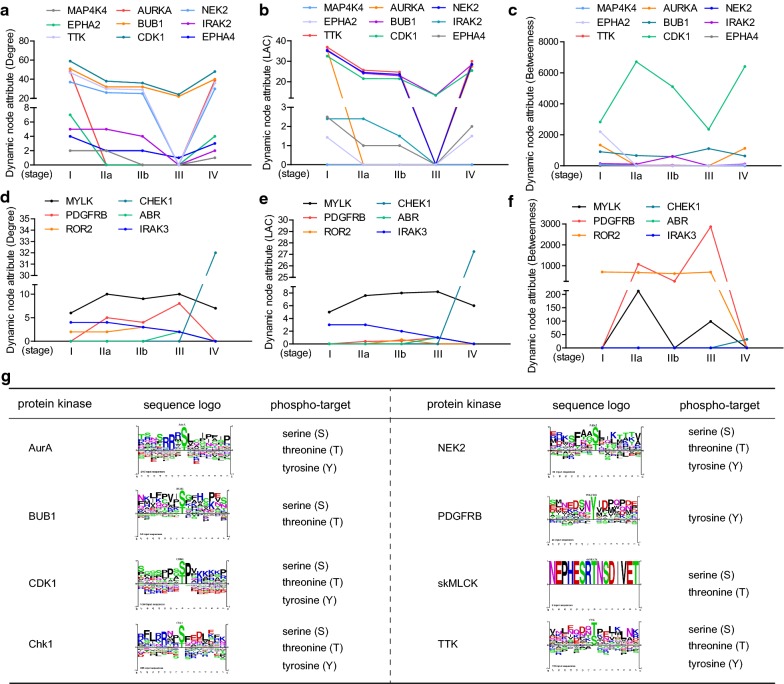
Identification of PDAC stage-specific activated kinases and their phosphosite markers. **a**–**f** Featured kinases with values of DC, LAC and BC from dynamic networks at different stages. **g** Phospho-target and sequence logo of protein kinases were predicted and listed

Considerable studies have shown a causal role of protein kinase mutations or dysregulations in tumorigenesis and cancer progression. Cancer research has been trying to turn these molecules into valid drug candidates for emergence targeted therapies. Depending on the dynamic networks, the PDAC stage-specific activated protein kinases were also identified. As shown in Fig. 9a–f, a total of 15 kinases involved in the dynamic networks with distinct patterns. TTK, AURKA, BUB1, CDK1 and NEK2 were fundamental kinases with high node degree, which may be required for maintaining the molecular signals underlying tumor progression (Fig. 9a). Besides, we also found CHEK1, the checkpoint kinase 1 coding gene (degree = 32), specifically activated at stage IV (Fig. 9d), which may indicate a potential drug target for advanced PDAC. The kinase ABR was the featured node for stage III, though getting low node degree (Fig. 9d). Additionally, PDGFRB was particularly found at stage IIa, IIb and III in the dynamic network (Fig. 9d). Finally, we also predicted the phospho-targets of eight candidate kinases. The protein kinases AurA (encoded by AURKA), CDK1, Chk1 (encoded by CHEK1), NEK2 and TTK phosphorylated their substrates by targeting serine, threonine and tyrosine, while BUB1 and skMLCK (encoded by MYLK) targeted the serine and threonine for phosphorylation (Fig. 9g). Tyrosine was the only phosphorylation site of PDGFRB substrates (Fig. 9g). Moreover, the consensus sequences of kinase substrates were depicted by sequence logo to indicate the phosphosite markers (Fig. 9g).

### Validation of gene expression patterns in PDAC tissues

**Fig. 10:**
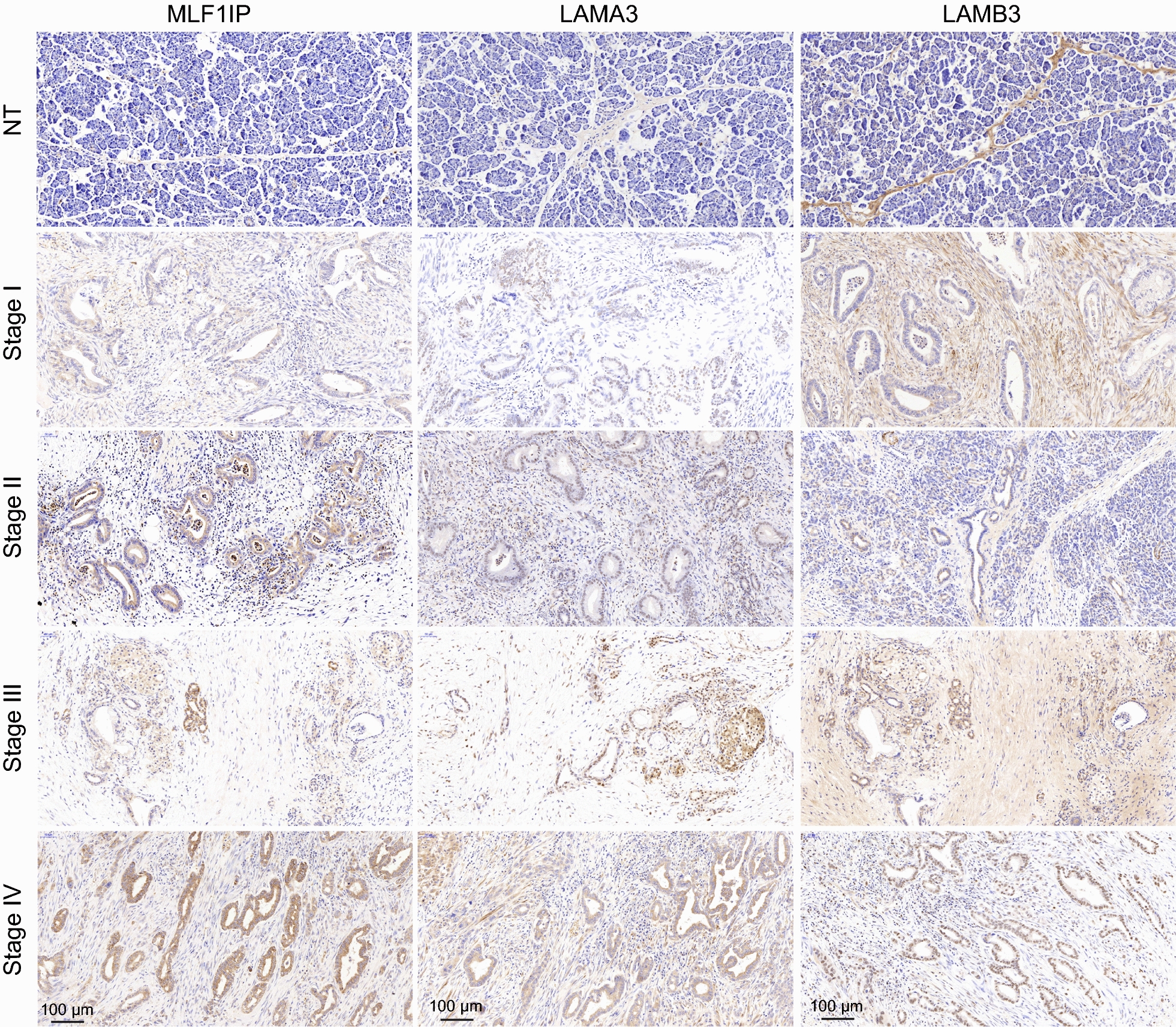
Experimental validation of gene expression patterns in PDAC tissues. Expression manners of MLF1IP, LAMA3 and LAMB3 in different PDAC staging tissues and normal pancreatic tissues were performed by immunohistochemistry

To investigate the expression patterns of MLF1IP, LAMA3 and LAMB3 during PDAC progression, we performed the immunohistochemistry (IHC) analysis. As a featured node of stage IV, MLF1IP expressed weekly in normal pancreatic tissue (Fig. 10). With the progression of PDAC, MLF1IP dramatically increased in PDAC tissues and was cytoplasmic and nuclear localization. LAMA3 and LAMB3 were common genes shared by five fundamental pathways throughout different PDAC stages. The IHC analysis showed that LAMA3 and LAMB3 were positively stained in PDAC tissues with a progressive increase manner (Fig. 10). Interestingly, we found that LAMB3 positively expressed in normal pancreatic connective tissue. With the initiation of PDAC, LAMB3 mainly existed in stroma and cytoplasm of cancer cells. However, LAMB3 gradually translocated into the nucleus of cancer cells at advanced PDAC stages. This special expression manner may indicate the pathologic molecular basis of tumorigenesis and progression.

## Discussion

PDAC is one of the most lethal tumors with limited survival improvement over the last decades. The rapid progression of PDAC results in an advanced stage of patients when diagnosed. Kong et al. [17] utilized inflammation-accelerated Kras^G12D^-driven PDAC mouse model to illustrate the dynamic landscape of pancreatic carcinogenesis. Their high temporal resolution transcriptional data defined a transcriptional signature of early pancreatic carcinogenesis and a molecular network driving formation of preneoplastic lesions. However, the dynamic molecular mechanism underlying PDAC progression remains far from clear. In this study, we analyzed the microarray from GEO and identified the DEGs between normal tissue and different staging PDAC, respectively. Further, we constructed a dynamic molecular interaction networks and identified the functional modules and featured nodes for each PDAC stage, which may be responsible for PDAC progression.

The molecular basis of different PDAC stages were complex and dynamic. The stage-course gene expression patterns profiled landscape of differences between PDAC stages. TNNT1, encoding slow skeletal muscle troponin T, kept increasing significantly from early to advanced stage. TNNT1 was up-regulated in human induced pluripotent stem cells and immortalized retinal pigment epithelial [18]. Studies demonstrated that TNNT1 involved in breast cancer cell proliferation and highly expressed in leiomyosarcoma metastases [19, 20], while its role in PDAC remained mystic. Li et al. [21] found that HOOK1 negatively regulated epithelial–mesenchymal transition by inhibiting the activity of SHP2. The dramatically decrease of HOOK1 in PDAC may suggest a molecular basis of aberrant EMT during cancer progression. Pathway enrichment showed that pathways in cancer, small cell lung cancer, ECM-receptor interaction, amoebiasis, focal adhesion were commonly enriched from early to advanced stage, which should be essential for maintaining the pathological status of PDAC. Except for amoebiasis, these pathways were significantly associated with poor PDAC overall survival. Further study suggested that LAMA3, LAMB3, LAMC2, COL4A1 and FN1 were commonly shared by these five key pathways and negatively correlated with overall survival. LAMA3, LAMB3 and LAMC2 encoded the subunits of laminin, which was component the basement membrane and involved in cell migration. Recently study demonstrated that combination of serum LAMC2, CA19.9 and CA125 was able to significantly improve upon the performance of CA19.9 alone in detecting PDAC [22], while the exact function of LAMC2 need to be fully elucidated. The role of LAMA3 and LAMB3 in PDAC were rarely studied, too. We found that LAMA3 and LAMB3 were robustly expressed at each PDAC stage when compared with normal tissue, which should be essential for tumorigenesis and progression.

There were also some pathways enriched at particular stage. Rheumatoid arthritis was specifically found at stage I and IIa, which indicated that immune dysregulation occurred early during tumorigenesis. A recent study from Ikeura et al. [23] also showed that autoimmune pancreatitis has the increased risk for pancreatic cancer after 62.4 months of mean follow-up period. Thus, the relationship between autoimmune and pancreatic cancer need to be closely concerned. For stage IV, three pathways including p53 signaling pathway, platelet activation, and cell cycle were specifically identified. Missense mutations in the p53 tumor suppressor inactivated its antiproliferative properties but could also promote metastasis through a gain-of-function activity [24]. Aberrant p53 signaling were predominately seen in some in situ lesions as well as invasive PDAC, indicating this signaling may occur mid-to-late stage in the pathogenesis of this disease [25]. Deregulation of cell cycle has also been implicated in PDAC progression. Six genes including CHEK1, CCNB1, CCNB2, CDK1, CDKN2A and SFN were shared by p53 signaling pathway and cell cycle. Due to the poor outcome of advanced PDAC, promising therapies like cell cycle inhibitors are currently under development [26]. Extravasated platelet activation in pancreatic cancer and stroma were associated with tumor metastasis [27]. Inhibition of platelet activation prevented the P-selectin and integrin-dependent accumulation of cancer cell microparticles and reduced tumor growth and metastasis [28]. Moreover, activated platelet that interacted with cancer cells was also sufficient to prime cisplatin insensitivity in pancreatic cancer cells [29]. In our study, we found a total of 12 genes were enriched in platelet activation, among which half of them were collagen family members (COL1A1, COL1A2, COL3A1, COL5A2, COL11A1). These results may indicate a novel role of collagens in facilitating PDAC development. However, collagen-mediated platelet activation during PDAC progression still need to be fully demonstrated.

The progression of PDAC exhibited dynamic molecular interaction networks from early to advanced stage, among which highly interconnected regions were considered as key regulators for maintaining molecular networks. We identified a total of 45 unique clusters for five stages, and these clusters showed special expression pattern at different stages. Pathways enrichment indicated that cell cycle, protein digestion and absorption and ECM-receptor interaction were fundamental signaling for all five PDAC stages. Moreover, the collagen family and integrins were two main regulators for protein digestion and absorption and ECM-receptor interaction. PDAC is characterized by the excessive deposition of extracellular matrix (ECM), which is thought to contribute to its malignant behavior. Duan et al. [30] found that type I collagen could promote epithelial–mesenchymal transition in pancreatic cancer by activating β1-integrin coupling with the Hedgehog pathway. In our study, we found high expression of integrin subunit beta 4 (ITGB4) in PDAC was correlated with poor prognosis. This result was in consistent with Masugi’s study [31], whose group noted that upregulation of integrin β4 promoted epithelial–mesenchymal transition and was a novel prognostic marker in PDAC. Intriguingly, we also found that ITGB4 typically contributed to molecular network of stage IV, which suggested a temporal dependent manner of ITGB4 for promoting PDAC progression. Due to the essential role of ITGB4 in advanced PDAC, the underlying mechanism need to be further elucidated.

In the dynamic molecular interaction network, topological variation of nodes was essential for network progress. Basing on the typical centrality measures, we found total 19 key nodes uniquely contributed to five stages, respectively. At early stage, aberrant cell mitosis and motility were frequently required for tumorigenesis. Featured nodes including NDC80, ZWINT, OIP5, KIF2C and KIF20A were mainly associated with chromosome segregation and spindle checkpoint activity. Overexpression of NDC80 was correlated with prognosis of pancreatic cancer and regulated cell cycle and proliferation [32]. With the progression of PDAC, tumor stroma became more and more abundant. As mentioned above, cancer cells actively involved in the production of extracellular matrix proteins and interacted with ECM by integrins. Integrins played a role in cell migration, morphologic development, differentiation, and metastasis. Node centrality analysis showed that ITGB1 (stage IIa), ITGA4 (stage III), ITGA9 (stage III), ITGB4 (stage IV) specifically functioned at particular stage, which indicated an essential role of integrins during PDAC progression. Moreover, a total of four key nodes were identified at stage IV, which may facilitate the aggressiveness of PDAC. The clinical relevance analysis suggested that MLF1IP (also known as CENPU) and ITGB4 were significantly correlated with shorter overall survival. Role of MLF1IP had been preliminarily elucidated in some cancers including bladder cancer, ovarian cancer and prostate cancer, while little is known in PDAC progression [33–35]. The precise function of MLF1IP in PDAC need to be further investigated.

Protein kinases have been widely investigated in cancers, since they are promising molecular targets for cancer treatment. Depending on the dynamic molecular interaction networks, several PDAC stage-specific kinases were identified. TTK, AURKA, BUB1, CDK1 and NEK2 were fundamental kinases that participated in cell cycle and mitosis. The AURKA selective inhibitor alisertib could induced cell cycle arrest and facilitated autophagic cell death in pancreatic cancer cells [36]. The phase I trial (NCT01924260) was carrying out to investigate the safety and efficacy of alisertib in pancreatic cancer patients when given in combination with gemcitabine. CHEK1 was required for checkpoint-mediated cell cycle arrest and preserving the integrity of the genome. Recent study pointed that patients with inactivating homologous recombination repair (HRR) related gene mutations showed significantly longer PFS than those without HRR-related gene mutations after oxaliplatin-based chemotherapy [37]. In our study, we found that CHEK1 was highly activated in stage IV, which may reveal the malignant evolution of PDAC and potential therapeutic target.

## Conclusions

In summary, we screened the DEGs of different PDAC stages and constructed dynamic molecular interaction network to illustrate the underlying mechanism of PDAC progression. Five genes that commonly shared by five fundamental pathways may act as key regulators throughout all PDAC stages. Meanwhile, collagen family and integrins were identified as potential regulators for driving PDAC progression. Additionally, PDAC stage-specific protein kinases were also identified for potentially targeted therapy. These timing and context dependent nodes and pathways could be pivotal mechanism for promoting the dynamic progression of PDAC. Our study provided a view for a better understanding of the dynamic landscape of molecular interaction networks during PDAC progression and offered potential opportunities for therapeutic intervention. Further studies such as single-cell sequencing or other multi-omics analysis are needed to comprehensively reveal the intricate mechanism of cancer cells and tumor microenvironment changes during PDAC progression.

## Additional file

**Additional file 1: Figures S1–S5.** Featured clusters at different PDAC stages.

## Abbreviations

PDAC: pancreatic ductal adenocarcinoma
DEGs: differentially expressed genes
logFC: log2 fold change
OS: overall survival
IHC: immunohistochemistry
GEO: Gene Expression Omnibus
GO: gene ontology
KEGG: Kyoto Encyclopedia of Genes and Genomes
PPI: protein–protein interaction
FDR: False Discovery Rate
TCGA: the Cancer Genome Atlas
DC: degree centrality
BC: betweenness centrality
LAC: local average connectivity-based method
ECM: extracellular matrix
