# Correction to: Analysis of dynamic molecular networks for pancreatic ductal adenocarcinoma progression

**DOI:** 10.1186/s12935-020-01329-1

**Published:** 2020-06-11

**Authors:** Zongfu Pan, Lu Li, Qilu Fang, Yiwen Zhang, Xiaoping Hu, Yangyang Qian, Ping Huang

**Affiliations:** 1 Department of Pharmacy, Zhejiang Cancer Hospital, Hangzhou, 310022 China; 2 Department of Pharmacy, The First Affiliated Hospital, College of Medicine, Zhejiang University, Hangzhou, 310003 China; 3 Key Laboratory of Head & Neck Cancer Translational Research of Zhejiang Province, Zhejiang Cancer Hospital, Hangzhou, 310022 China

## Correction to: Cancer Cell Int (2018) 18:214 10.1186/s12935-018-0718-5

Following publication of the original article [1], the authors notified us that Fig. 3a was incorrect.

The graph presented in Fig. 3a is the same as Fig. 2a in the published manuscript. This was done erroneously during the prep for the manuscript. The figure below represents the true migration values achieved for cells blocked in interphase and treated with the different compounds.

The corrected graph in Fig. 3a is presented below.
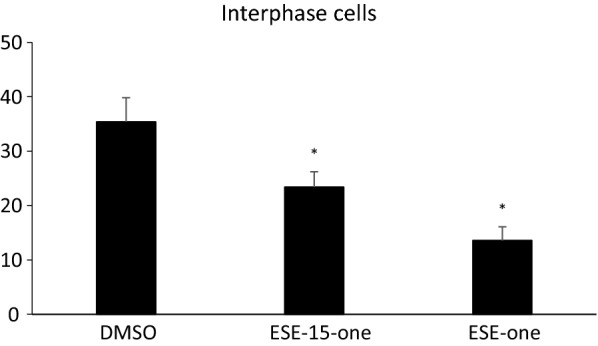


Specifically, blocked cells treated with DMSO closed 35% of the wound while ESE-15-one reduced that to 23% and ESE-one reduced this to 13%. T-tests show statistical significance
