# Supplementary material for: Analysis of dynamic molecular networks for pancreatic ductal adenocarcinoma progression
Source: Cancer Cell Int. 2018 Dec 22;18:214. doi: 10.1186/s12935-018-0718-5 (PMC6303882; doi:10.1186/s12935-018-0718-5)
Supplement: Supplementary file 1 — Additional file 1: Figures S1–S5. Featured clusters at different PDAC stages. [file 12935_2018_718_MOESM1_ESM.pdf]

Featured clusters at different PDAC stages

The networks from five stages were input into DyNetViewer and calculated by MCODE algorithm over time. A total of 45 featured molecular modules were identified for all PDAC stages. The number of featured clusters for each stage were 11 (stage I), 10 (stage IIa), 10 (stage IIb), 9 (stage III) and 8 (stage IV), respectively (Fig. S1a, S2a, S3a, S4a, S5a). The dynamic changes of featured modules were illustrated over time (Fig. S1b, S2b, S3b, S4b, S5b) (charts were also shown in Fig. 5a-e).

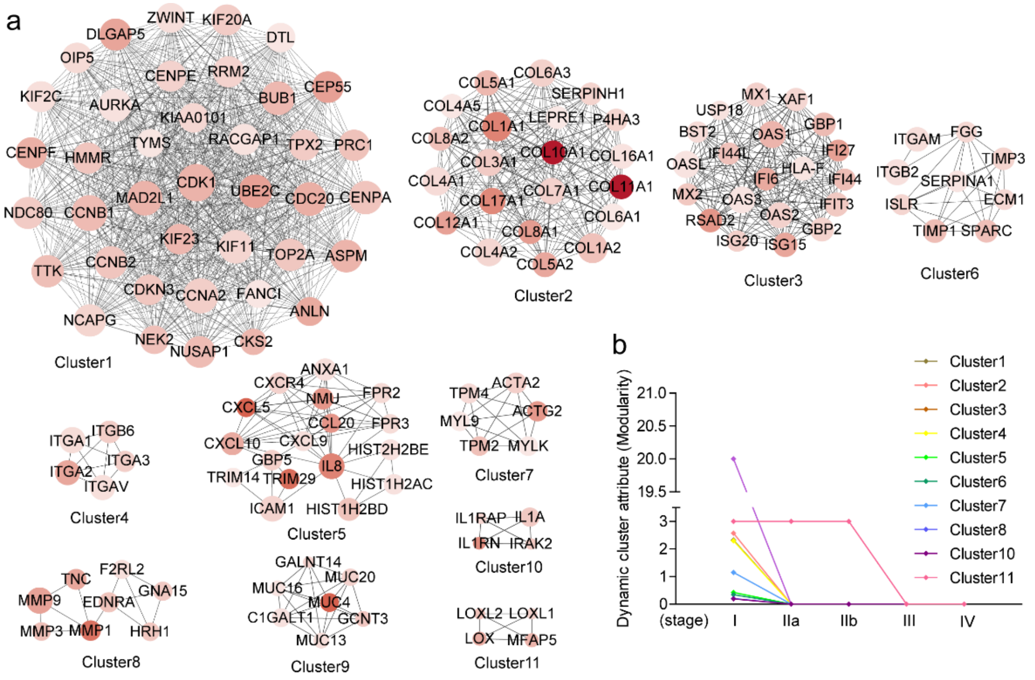

Figure S1 Clusters analysis of dynamic network at stage I. **a** The list of featured clusters identified from dynamic network at stage I. **b** The dynamic charts of featured clusters attributes identified from dynamic networks. The dynamic cluster attributes for five stages were calculated by DyNetViewer (MCODE algorithm) over time.

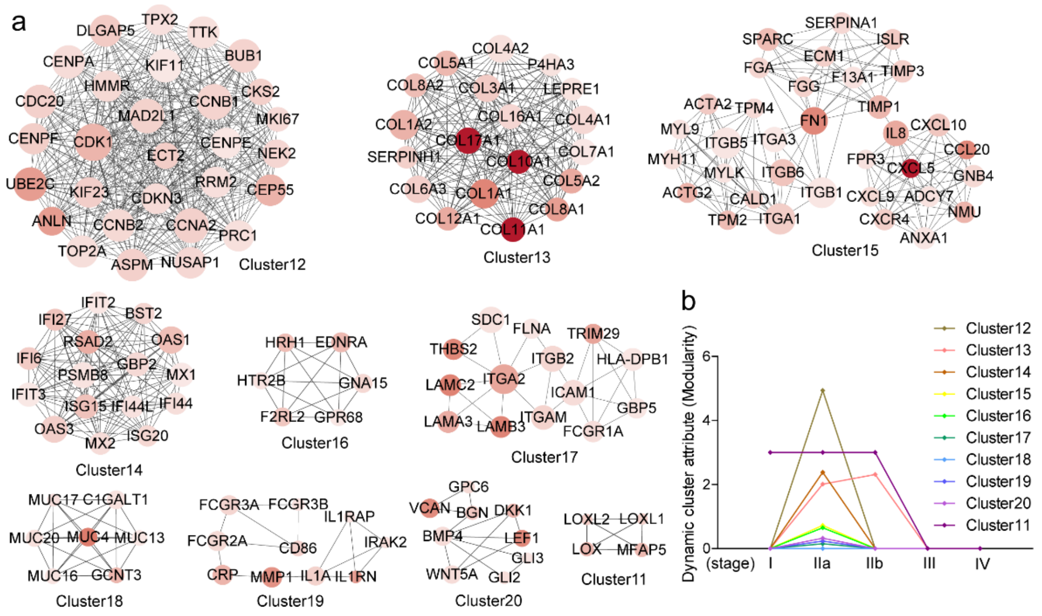

Figure S2 Clusters analysis of dynamic network at stage IIa. **a** The list of featured clusters identified from dynamic network at stage II. **b** The dynamic charts of featured clusters attributes identified from dynamic networks.

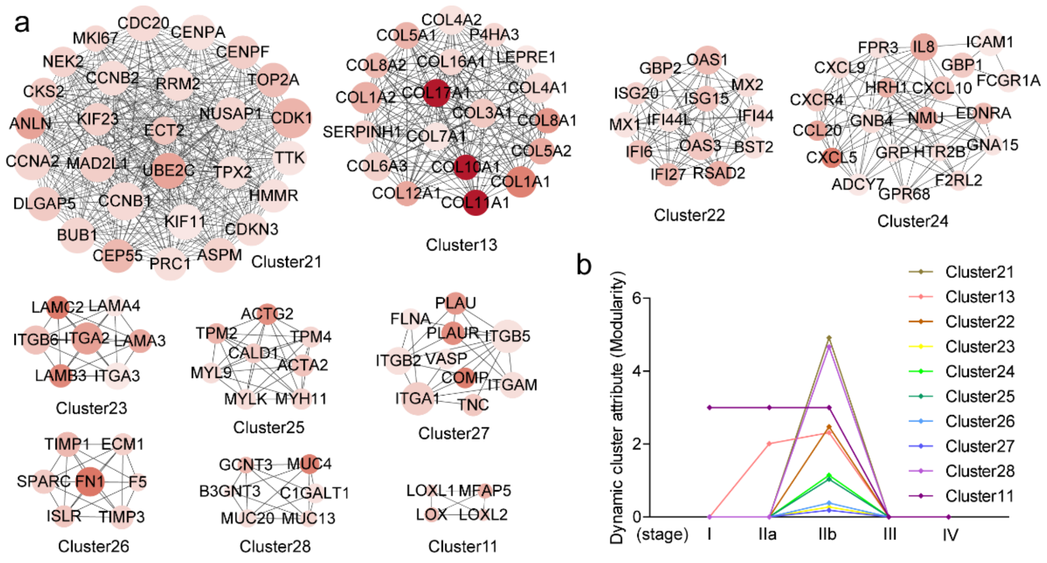

Figure S3 Clusters analysis of dynamic network at stage IIb. **a** The list of featured clusters identified from dynamic network at stage IIb. **b** The dynamic charts of featured clusters attributes identified from dynamic networks.

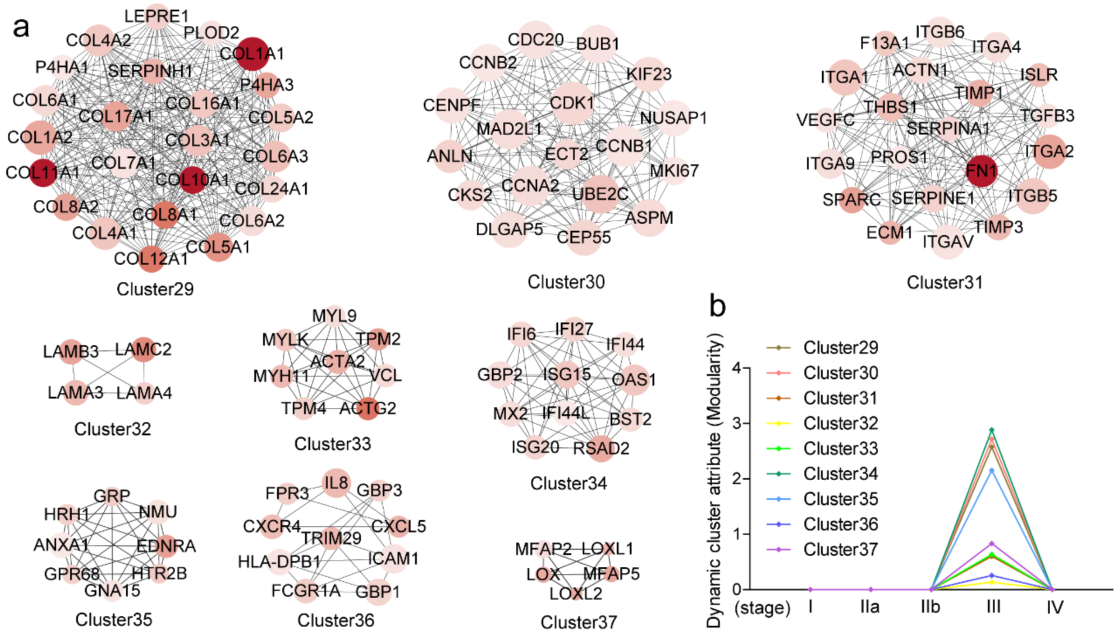

Figure S4 Clusters analysis of dynamic network at stage III. **a** The list of featured clusters identified from dynamic network at stage III. **b** The dynamic charts of featured clusters attributes identified from dynamic networks.

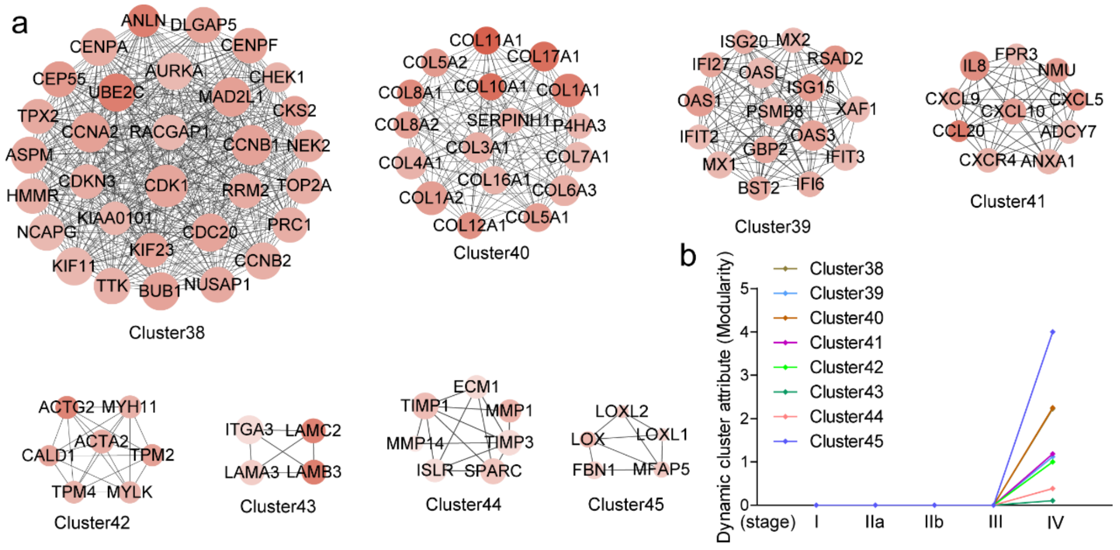

Figure S5 Clusters analysis of dynamic network at stage IV. **a** The list of featured clusters identified from dynamic network at stage IV. **b** The dynamic charts of featured clusters attributes identified from dynamic networks.
